# On the Move: Trajectories of Stressors and Rewards Among Relocating Couples

**DOI:** 10.1177/01461672251355002

**Published:** 2025-08-03

**Authors:** Hanieh Naeimi, Haeyoung Gideon Park, Matthew D. Johnson, Mariko L. Visserman, Rebecca Horne, Emily A. Impett

**Affiliations:** 1 University of Toronto, ON, Canada; 2 University of Alberta, Edmonton, Canada; 3 University of Sussex, Mississauga, ON, Canada; 4 Department of Product Architecture and Operations, CrowdStrike, Toronto, Canada

**Keywords:** relocation, close relationships, stress, couples, dyadic latent growth curve

## Abstract

Partnered relocation—when couples move to support one partner’s career—is increasingly common and involves unique stressors and rewards. In a longitudinal study of 206 couples (*N* = 383, 177 dyadic, 29 individual reports) surveyed 2-months pre-relocation and 3-, 6-, 9-, and 12-months post-relocation, we examined how stressors and rewards change over time, comparing experiences of partners who initiate (relocators) versus support the move (accompanying partners). Using dyadic latent growth curve modeling of stressors and rewards across 12 domains (e.g., careers, social networks, living arrangements, and logistics), we found that most stressors declined, and several rewards increased over time, with some differences between relocators and accompanying partners. We also explored the role of gender, COVID timing, move distance, socioeconomic status, and relationship satisfaction as covariates of the trajectories. This study highlights how couples adapt during relocation depending on relational and contextual factors.

In today’s globalized world, relocation for work is increasingly common. From 2017 to 2022, over half a million Canadian households moved for job opportunities (Statistics Canada, 2022), and since 2020, 5 million Americans have relocated due to remote work (Flynn, 2023). Relocation can be stressful, especially for couples where one partner’s job is the reason for the move but both partners need to adjust their lives and careers (Brown, 2008; Challiol & Mignonac, 2005). Couples’ relocation can take a toll on individuals’ well-being (Anderzén & Arnetz, 1997; Brown, 2008; Munton, 1990; Riemer, 2000) and leave couples vulnerable to dissatisfaction and divorce (Sweatman, 1999; McNulty, 2015).

Despite the frequency and relational significance of partnered relocation, most research has relied on cross-sectional data, offering limited insight into how couples adjust over a longer period of time (Sterle et al., 2018). Moreover, research has focused almost exclusively on the challenges and difficulties of relocation, such as feelings of isolation and strain on relationships (Brown, 2008). Positive aspects have been overlooked, such as factors that may have motivated people to move in the first place (e.g., better financial opportunities, career growth; Challiol & Mignonac, 2005) or rewarding aspects that people did not initially anticipate (e.g., exploring a new culture or learning a new language; Harris, 2004). It is essential to consider a diverse range of stressors *and* rewards people may experience during relocation, and how these may co-exist, to provide a balanced view of this transition. To address these gaps, the current study offers a more comprehensive understanding of how stressors and rewards change over time, from 2 months before and across a 1-year period after relocation. We also consider the impact of relocation for partners who initiate the move and those who accommodate their partner, and explore whether gender, COVID-19, distance moved, socioeconomic status, and relationship satisfaction shape these trajectories.

## Relocation Stressors and Rewards

Relocation is a complex life transition that involves significant change and adaptation. Relocation presents a range of demands—adjusting to a new environment, establishing new social ties, managing logistics—that can create significant changes in people’s lives and lead to prolonged stress (Brown, 2008; Harvey, 1998). Indeed, people who relocate for work (e.g., expatriates, international businesspeople), as well as their accompanying partners, have reported elevated levels of stress throughout this transition (Brown, 2008; Harris, 2004; Harvey, 1998; McNulty, 2012). Commonly cited stressors include starting a new job, moving away from family, losing old or forming new relationships, experiencing changes in lifestyle or standard of living, and adapting to a new culture or language (Munton, 1990).

However, most studies have examined these stressors cross-sectionally, providing only a snapshot of individuals’ experiences of stress rather than how these experiences fluctuate over time. Given that relocation-related stress is often acute (i.e., with a clear onset and the possibility of an endpoint; Karney & Neff, 2013), people may adjust to their new environment as the transition progresses (Brown, 2008). However, typical trajectories of perceived stress over the course of relocation are unknown. Given that individuals often adjust to their circumstances (Lucas et al., 2003), understanding how stress fluctuates across the relocation process is essential.

While prior research has almost exclusively focused on negative experiences during relocation, the presence of negative experiences does not necessarily imply the absence of positive ones. Importantly, negative and positive experiences are not mutually exclusive and often coexist. For instance, starting a new job or moving into a new home can be both stressful and rewarding—stressful due to uncertainty or disruption, and rewarding due to novelty and growth. Although relationship research has historically treated positive and negative experiences as opposing ends of a continuum (Don et al., 2024), findings from other fields suggest that positive and negative experiences often function as distinct dimensions (Farrell et al., 2018). For instance, emotion research conceptualizes positive and negative affect as separate constructs rather than as inversely related (Watson et al., 1988). Similarly, studies on optimism and pessimism (Marshall et al., 1992), as well as emerging research on ambivalence (Righetti et al., 2020; Schneider & Schwarz, 2017), suggest that positive and negative evaluations can coexist, with each requiring distinct measurement and analysis. These frameworks suggest that to fully understand people’s experiences, it is essential to examine both positive and negative evaluations separately.

In fact, although relocation is often characterized as stressful, individuals also report positive aspects of relocation, such as learning about a new culture or experiencing personal growth (Riemer, 2000). More broadly, engaging in novel experiences has been linked to personal growth and self-expansion, which, despite their challenges, can be inherently rewarding (Aron et al., 2000; West et al., 2024). Although stress levels are often heightened at the time of relocation, they may decline over time as individuals gradually adapt to their new environment. Conversely, the presence of positive experiences and rewards may be relatively low during the initial transition, when uncertainty and disruption dominate, but may increase as individuals settle into their new roles, communities, and routines. This pattern is consistent with adaptation models suggesting that people tend to recover from initial disruptions and reestablish equilibrium over time (Lucas et al., 2003). For example, while the early stages of relocation may be characterized by logistical burdens, social isolation, and job-related stress, later stages may involve a greater appreciation for new opportunities, stronger connections, and a sense of mastery over challenges.

Building on these insights, our study examines how both stress and reward evolve over time in tandem across 12 domains, grouped into four overarching categories: careers, social networks, living arrangements, and logistics. These domains, identified in previous relocation literature (Brown, 2008; Munton, 1990), capture both the practical and relational aspects of the relocation experience.

## Relocation Role and Gender

While over two-thirds of relocations involve a romantic partner (Brookfield Global Relocation Services, 2016), past studies have primarily focused on either relocators (Holopainen & Björkman, 2005; Kempen et al., 2015) or accompanying partners (Cole, 2012; Lauring & Selmer, 2010), or did not clearly account for both partners’ experiences even when examined simultaneously (Brown, 2008; Coulter et al., 2012; Harvey, 1998; Munton, 1990; Riemer, 2000). Only one study has examined both partners’ experiences during relocation while considering the interdependence of partners’ reports to study the role of couples’ personality in their adjustment (van Erp et al., 2014). Past work has noted that the most common reasons for an unsuccessful relocation are accompanying partners’ career concerns (Lazarova et al., 2015) and their difficulties with adapting to a new environment (Haslberger & Brewster, 2008).

Other studies have highlighted discrepancies in the concerns expressed by relocators and accompanying partners before and after the move (Harvey, 1998). For instance, before the move, relocators reported being most concerned about their job performance and career stage, while accompanying partners reported being most concerned about family stress and disruption to their careers (Harvey, 1998). Relatedly, after the move, relocators may have an easier time adjusting since the continuance of their job provides stability, whereas accompanying partners often need to start anew, especially if they lost their job because of the move (Harvey, 1998; Hausman & Reed, 1991). On the other hand, relocators may carry the burden of guilt and responsibility for changes made to the couple’s lives, which can create additional stress. Hence, it is crucial to examine the experiences of relocators and accompanying partners within a dyadic framework, recognizing how each partner’s role in the relocation process may shape trajectories of stressors and rewards. By understanding these interdependent experiences, we can better support couples as they navigate the challenges and opportunities of relocation, allowing both individuals and institutions to respond more effectively to the needs of each partner.

Moreover, traditional gender roles often shape these experiences. Past studies have disproportionately examined women as accompanying partners (Challiol & Mignonac, 2005), framing dual-career couples as exceptions (Harvey, 1998; McNulty, 2012, 2014). Yet, these societal dynamics are shifting, with women initiating relocation and more men assuming a supportive role. These shifts raise new questions about how patterns of stress and reward are shaped by gender and a person’s role in the relocation. For example, relocating women (vs. relocating men) experience greater stress during relocation such as increased demands adjusting to a new job while continuing to take on domestic chores (Harris, 2004) and more work–life conflict (Mäkelä et al., 2017). Relatedly, past work has found that accompanying men (vs. accompanying women) feel more isolated and need more support (Cole, 2012). Recognizing how both role and gender shape relocation experiences can help researchers and practitioners more effectively support the well-being of diverse couples navigating relocation.

## Overview of Research

The current study utilizes a sample comprising relatively equal numbers of relocating men and women. To better understand the trajectories of our key variables over time, we explore how the experiences of 12 variables related to 4 categories of stressors and rewards (careers, social networks, living arrangements, and logistics) change from 2 months before and across the first year after relocation for relocators and accompanying partners and how gender influences these trajectories. To contextualize the heterogeneity in experiences, we examine gender, COVID-19, distance moved, socioeconomic status, and relationship satisfaction as covariates.

## Method

### Participants and Procedure

We collected data from 227 couples and 1 individual (*N* = 455) who were planning on relocating for a partner’s job or schooling in the near future through multiple recruitment platforms (e.g., Reddit forums dedicated to relocation processes, email listservs for professionals in the academic community, Kijiji/Craigslist, relocation forums, Facebook pages and alumni groups, the senior author’s lab website, Respondent, and Society for Personality and Social Psychology). Data collection started in August 2019 and was completed in September 2022. Due to a part of our data collection taking place during the COVID-19 pandemic, we included COVID-related timing as a covariate in our analyses.

Couples were eligible to participate if both partners spoke English, were over the age of 18, were in a romantic relationship, currently lived together, and intended to relocate with their partner in at least 2 months, which was primarily for one of the partners (e.g., to support their career opportunities for school or work). Interested couples were enrolled after a phone interview to confirm their eligibility. This study was inclusive to all individuals regardless of their geographical location, gender identity, biological sex, and sexual orientation. See Table 1 for detailed demographic information. To complete our longitudinal analyses, we required participants to have completed at least two out of five surveys (*N* = 383; 177 dyadic, 29 individual reports). All participants completed the baseline survey approximately 2 months before relocating, along with follow-up surveys at 3 months (*N* = 365), 6 months (*N* = 347), 9 months (*N* = 326), and 12 months (*N* = 288) after relocation. Some attrition was due to couples breaking up over the course of the study (*N* = 38, 8.3%). The materials and codebook are publicly available on the Open-Science Framework (OSF; https://osf.io/79rqd/ ) and The Love Consortium Dataverse (https://doi.org/10.15139/S3/NOZYUD ).

**Table 1.** Sample Demographics (*N* = 457).

Variable	Mean	Standard deviation
Age	29.8	5.8
Relationship Length	6.26 years	4.98 years
Socioeconomic Status (out of 10)	6.25	1.6
Distance moved (in kms)	2,702	3,195
	*n*	%
Type of Move		
To a different city	107	23.6
To a different province/state	210	46
To a different country	136	29.8
Move Reason		
Partner’s work	128	33.6
Partner’s school	51	13.4
Own work	125	32.8
Own school	56	14.7
Wanted to live somewhere new	5	1.3
Other	10	2.6
Origin Location by Continent		
Africa	4	.88
Asia	16	3.5
Europe	66	14.5
North America	359	78.9
Oceania	2	.44
South America	8	1.76
Own Race/Ethnicity		
Bicultural/Multicultural	21	4.6
Black	34	7.5
East Asian	40	8.8
Latin American	19	4.2
Native American	3	.7
South Asian	37	8.1
White	286	62.9
Other	15	3.3
Have Children		
Yes	105	23.1
No	350	76.6
Gender		
Man	212	46.6
Woman	232	51.0
Non-Binary	9	2.0
Other	2	.4
Sexual Orientation		
Asexual	20	2.2
Bisexual	36	7.9
Gay	8	1.8
Heterosexual	364	80
Lesbian	15	3.3
Pansexual	8	1.8
Queer	10	2.2
Other	4	.9
Relationship Status		
Dating	125	27.5
Common-law	65	14.3
Engaged	42	9.2
Married	216	47.5
Other	7	1.5

### Measures

#### Stressors and Rewards

Stressors and rewards were collected at baseline and in all follow-up surveys. To measure the overall stress of relocation, participants answered a question about how stressful they were finding the relocation process in general (adapted from Martin, 1996). Then, participants answered how negative (e.g., stressful, upsetting) they found 12 different stressors in relation to their current relocation process^1^ (adapted from Munton, 1990). For clarity of presentation, we discuss these domains under four broad categories: (a) *Careers*: starting a new job/school, partner’s job/school; (b) *Social networks*: moving away from family, moving near family, self/partner losing social ties, establishing new social ties; (c) *Living arrangements*: finding a new place of residence, settling into a new home/neighborhood, changes in standard of living; and (d) *Logistics*: finances related to the move, administrative tasks, and navigating a new culture/language. All items were rated on a 7-point scale (1 = *not at all stressful* to 7 = *very stressful*). Using these same 12 items, participants also rated how positive (e.g., rewarding, exciting) they found the specific areas in relation to their current relocation process (1 = *not at all rewarding* to 7 = *very rewarding*). We also calculated bivariate correlations between the extent to which participants rated each domain (e.g., starting a new job/school) as stressful and rewarding, separately for each role (relocating and accompanying partner) across each time point (baseline, 2-months pre-relocation and 3-, 6-, 9-, and 12-months post-relocation). Most correlations were non-significant, and all but one were less than *r* = .42 (see Table 2), which lends credence to our decision to model separate trajectories of stressors and rewards.

**Table 2.** Bivariate Correlations Between Relocators’ and Accompanying Partners’ Own Reports of Each Stressor and Reward at Each Time Point from baseline to 12 Months After the Move.

Variable	Relocator					Accompanying partner				
	Baseline	Time 1	Time 2	Time 3	Time 4	Baseline	Time 1	Time 2	Time 3	Time 4
Careers										
Starting a new job/school	.02	−.001	−.05	−.02	−.14	−.02	−.07	−**.21**	−**.26**	−**.27**
Partner’s job/school	.08	−.12	−.10	−.14	−.03	.14	.07	−.02	−.03	−.14
Social Networks										
Moving away from family	−.11	−.09	−**.24**	−.13	−.09	−.08	−.07	−.06	−**.22**	−.17
Moving near family	−.18	.07	−.19	−**.29**	−**.61**	−**.26**	−.05	−.17	−**.37**	−**.31**
Self/partner losing social ties	.11	−.14	−.10	.04	−**.27**	−.005	−.11	−.14	−.13	.06
Establishing new social ties	.07	.01	−**.16**	−.16	−**.21**	.03	−.07	−.14	−**.21**	−**.21**
Living Arrangements										
Finding a new place of residence	.07	−.03	.02	.09	−.007	−.01	.02	−.06	−.14	−.09
Settling into a new home	.07	−.08	.01	.02	−.11	.004	−.11	−.13	−.12	−**.18**
Changes in standards of living	−**.26**	−**.31**	−**.31**	−**.32**	−**.30**	−**.27**	−**.32**	−**.29**	−**.33**	−**.42**
Adjustment										
Navigating a new culture	.12	**.26**	**.22**	.05	.06	.06	**.20**	.04	.09	−.07
Finances related to moving	−**.27**	−**.29**	−**.27**	−**.26**	−**.23**	−.01	−**.20**	−**.18**	.02	−.05
Admin tasks	−.007	−**.21**	−.08	−.04	−.16	.06	−.09	−.12	.05	.06

*Note.* Bolded coefficients are significant at *p* < .05.

### Covariates

Demographic information was collected in the baseline survey. Participants reported their socioeconomic status (SES) compared to other people in their country’s society on a 10-point scale (1 = *people who are the worst off, those who have the least money, least education, and worst jobs or no job*, 10 = *people who are best off, those who have the most money, most education, and best jobs*; Adler et al., 2000). We also coded whether participants were moving to a new city, state/province, or country. At baseline and during each follow-up, participants answered 18 items about several positive indicators of relationship quality, including satisfaction, commitment, intimacy, trust, passion, and love, from the Perceived Relationship Quality Component Inventory (Fletcher et al., 2000). An example item is “How satisfied are you with your relationship?” on a 7-point scale (1 = *not at all* to 7 = *extremely; M*_sample overtime_ *=* 5.90, *SD*_sample overtime_ *=* 0.75).

## Data Analytic Plan

We preregistered our analysis plan and conducted a power analysis prior to analyzing the data (https://osf.io/w4t2m/). We deviated from this preregistration in three ways: (a) We initially preregistered that we would model trajectories with only the follow-up datapoints but later recognized that we had measured all variables of interest at baseline (2 months prior to the move). We decided to include baseline reports in our trajectories to provide a more comprehensive assessment of trajectories from pre- to post-relocation; (b) We conducted exploratory analyses to test whether couples found each domain more stressful versus rewarding, and (c) We added several covariates in response to reviewer comments to better contextualize the heterogeneity in experiences of stressors and rewards.

We conducted all focal analyses with Mplus version 8.6 (Muthén & Muthén, 2015). As a first step, we ran unconditional univariate latent growth curve models separately for each partner (relocators and accompanying partners) for all variables, including stressors (i.e., general stress and individual stressors) and rewards. Intercept loadings in each model were set to 0, corresponding to initial levels at baseline (i.e., 2 months before the move), and linear slope loadings signified the passage of time based on the number of months that had passed since baseline data collection (0, 5, 8, 11, and 14). Nonlinear patterns were considered with latent basis growth models and quadratic models (McArdle & Epstein, 1987). For the latent basis growth models, we allowed the slope loadings to be estimated by the data (first and last measurement occasion loadings were set at 0 and 14, respectively, to identify the model). Since this approach does not have a functional form (e.g., quadratic, cubic), it is able to capture any non-linear pattern. We also examined quadratic models, which follow a set curved pattern. Each construct was fitted to a series of increasingly complex growth models (e.g., fixed intercept, random intercept, and fixed linear slope), and the change in model chi-square was used to determine the best-fitting trajectory.

Once the best-fitting trajectory was identified, we computed a series of dyadic latent growth curve models within a structural equation model (Kenny et al., 2006) for relocators and accompanying partners, one for each focal construct (general stress, 12 stressors, and 12 rewards) for a total of 25 models. Role (relocating vs. accompanying partner) was tested as a moderator by applying equality constraints to intercepts and then slopes when corresponding slopes reflected the same trajectory shape (e.g., both were linear, or both were curvilinear). In cases where the univariate growth curves revealed a linear slope for one partner and a curvilinear slope for the other, we were unable to directly compare the slopes between relocating and accompanying partners. Alternately for these cases, we tested intercept differences by setting the intercept at baseline (2 months pre move) and applying equality constraints across role. Similarly, we also set the intercept at the 12-months post move follow-up and applied equality constraints across role. In doing so, we determined whether initial and final levels significantly differed for relocators and accompanying partners, providing evidence as to whether their trajectories differed or not (Johnson et al., 2019; Little, 2023). Note that a significant worsening in the model chi-square (i.e., a χ^2^_diff_(1) > 3.84) after the application of equality constraints provided evidence of moderation, which allowed us to examine possible differences in trajectories between relocators and accompanying partners.

To better understand the heterogeneity in the trajectories, we examined several covariates including gender, move date in relation to the height of COVID-19, whether the move was international (vs. domestic), each person’s own SESand relationship quality. We accounted for the impact of relocators’ gender, COVID-19 (using relocation timing, pre- vs. post-January 1, 2021, when vaccines were first widely released in North America), whether the relocation was international, and each person’s SES at baseline. Additionally, each person’s own relationship quality was aggregated across all time points and entered as a covariate of their own trajectories (i.e., relocators’ relationship quality predicting their trajectory, accompanying partners’ relationship quality predicting their trajectory). We used the average relationship quality across waves to capture each participant’s general level of relationship functioning during the relocation period. This allowed us to examine how individuals with higher (vs. lower) average relationship quality during the transition differed in their trajectories of stressors and rewards, rather than relying on a single time point (e.g., pre-move or post-move) that may not adequately represent their broader relational experiences during this period. All analyses were conducted regardless of the type of relationship (mixed-gender or same-gender). Although our initial trajectories included people of all genders, we had to exclude seven participants who identified as non-binary from models with covariates.

Good global model fit was indicated by: (a) a non-significant chi-square value, (b) values of the confirmatory fit index and Tucker–Lewis index greater than 0.95, and (c) values of the root-mean-square approximation of error and the standardized root-mean-square residual less than 0.05. Model comparisons were made with chi-square difference testing where a χ^2^_diff_(1) > 3.84 indicated a significant worsening in model fit (Little, 2023). The influence of covariates on the trajectories was determined by *p* value significance testing with *p* < .05 indicating a significant association.

## Results

First, we conducted exploratory analyses to test whether couples found each domain more stressful versus rewarding by examining differences between average levels of stressors and rewards at each time point averaged across partners (see Table S1). On average, participants found moving away from family, losing social ties, relocation finances, and administrative tasks to be more stressful than rewarding. However, all other experiences—including starting a new position, moving near family, establishing new social ties, experiences with finding a new place, settling into a new home, standards of living, and navigating a new culture—were more rewarding than stressful. See Table 3 for means and standard deviations of all stressors and rewards across time points. Mean estimates for intercepts and slopes of the best-fitting model for all variables are reported in Table 4. All model fit statistics are posted on the OSF.

**Table 3.** Means and Standard Deviations of Stressors and Rewards From 2 Months Before to 12 Months After the Move, Aggregated Across Role.

Variable	Stressors					Rewards				
	2 Months before	3 Months after	6 Months after	9 Months after	12 Months after	2 Months before	3 Months after	6 Months after	9 Months after	12 Months after
General Stress	4.40 (1.57)	3.50 (1.69)	3.14 (1.64)	3.21 (1.64)	3.15 (1.67)	—	—	—	—	—
Careers										
Starting a new job/school	4.68 (1.88)	4.19 (1.96)	4.09 (1.93)	3.91 (1.98)	4.04 (1.92)	5.83 (1.37)	5.37 (1.56)	5.45 (1.57)	5.50 (1.41)	5.55 (1.49)
Partner’s job/school	3.71 (1.96)	3.54 (1.88)	3.62 (1.91)	3.78 (1.94)	3.70 (1.93)	5.42 (1.70)	5.00 (1.65)	5.03 (1.82)	5.08 (1.55)	5.18 (1.65)
Social Networks										
Moving away from family	4.05 (2.01)	3.69 (1.91)	3.87 (1.94)	3.92 (1.87)	3.76 (1.89)	2.72 (1.85)	3.09 (1.99)	2.90 (1.83)	3.05 (1.86)	3.17 (1.95)
Moving near family	2.89 (1.91)	2.94 (1.90)	2.96 (1.83)	3.08 (1.97)	3.15 (1.94)	4.89 (1.86)	4.84 (1.79)	4.54 (1.91)	4.72 (1.80)	4.69 (1.79)
Self/partner losing social ties	4.24 (1.91)	4.18 (1.79)	4.25 (1.77)	4.22 (1.81)	4.20 (1.89)	2.10 (1.54)	2.30 (1.71)	2.32 (1.64)	2.32 (1.56)	2.33 (1.64)
Establishing new social ties	4.16 (1.88)	4.23 (1.84)	4.14 (1.89)	4.08 (1.77)	4.12 (1.80)	5.27 (1.51)	4.84 (1.62)	4.73 (1.74)	4.76 (1.70)	4.97 (1.68)
Living Arrangements										
Finding a new place of residence	4.61 (1.80)	4.22 (1.95)	3.97 (1.95)	3.97 (1.90)	3.99 (1.91)	5.21 (1.53)	5.05 (1.58)	4.89 (1.68)	5.04 (1.59)	5.07 (1.63)
Settling into a new home	4.01 (1.78)	3.68 (1.68)	3.48 (1.70)	3.50 (1.73)	3.62 (1.80)	5.30 (1.43)	5.11 (1.37)	4.95 (1.48)	4.97 (1.50)	5.04 (1.54)
Changes in standards of living	3.58 (1.88)	3.15 (1.87)	3.07 (1.85)	3.05 (1.86)	3.09 (1.91)	4.69 (1.73)	4.63 (1.80)	4.58 (1.83)	4.63 (1.77)	4.71 (1.81)
Adjustment										
Navigating a new culture	3.54 (2.01)	3.39 (2.09)	3.47 (1.97)	3.47 (1.99)	3.34 (2.01)	4.97 (1.84)	4.40 (1.93)	4.35 (1.83)	4.32 (1.69)	4.34 (1.72)
Finances related to moving	4.44 (1.84)	4.23 (1.84)	4.12 (1.86)	3.92 (1.87)	3.93 (1.93)	3.21 (1.85)	3.14 (1.89)	3.33 (1.82)	3.47 (1.86)	3.56 (1.80)
Admin tasks	4.24 (1.77)	4.13 (1.80)	3.87 (1.85)	3.96 (1.81)	3.95 (1.82)	2.92 (1.74)	2.99 (1.87)	2.88 (1.77)	3.04 (1.84)	3.03 (1.78)

*Note*. Values outside parentheses are means and values inside parentheses are standard deviations. Items are rated on a scale from 1 to 7.

**Table 4.** Intercepts and Slopes of Stressors and Rewards from 2 Months Before to 12 Months After the Move.

Variable	Stressors						Rewards					
	Relocator			Accompanying partner			Relocator			Accompanying partner		
	Intercept	Slope	Quadratic	Intercept	Slope	Quadratic	Intercept	Slope	Quadratic	Intercept	Slope	Quadratic
General Stress	4.40^a^	−.08	—	4.40^a^	−.09	—	—	—	—	—	—	—
Careers												
Starting a new job/school	4.63^b^	−.04	—	4.63^b^	−.06	—	6.07	−.10	.005	5.39	−.07	.005
Partner’s job/school	3.74	—	—	3.35	.02	—	4.73	.006	—	5.83	−.04	—
Social Networks												
Moving away from family	4.11	−.08	.004	3.71	—	—	2.65^g^	.04	—	2.65^g^	.03	—
Moving near family	2.77^c^	.02	—	2.77^c^	.002	—	4.64^h^	—	—	4.64^h^	−.01	—
Self/partner losing social ties	4.28	—	—	4.09	—	—	2.16	.02	—	2.19	—	—
Establishing new social ties	4.11^d^	.005	—	4.11^d^	—	—	5.25^i^	−.03	—	5.25^i^	−.03	—
Living Arrangements												
Finding a new place of residence	4.69	−.13	.006	4.37	−.04	—	4.94	—	—	5.28	−.02	—
Settling into a new home	3.90	−.07	.004	4.09	−.05	—	5.29	−.02	—	5.29	−.03	—
Changes in standards of living	3.46^f^	−.03	—	3.46^f^	−.03	—	4.60^j^	—	—	4.60^j^	.004	—
Adjustment												
Navigating a new culture	3.19	—	—	3.19	−.02	—	4.82^k^	−.03	—	4.82^k^	−.05	—
Finances related to moving	4.50	−.04	—	4.35	−.04	—	3.01	.04	—	3.34	—	—
Admin tasks	4.31	−.03	—	3.95	—	—	2.94^l^	—	—	2.94^l^	—	—

*Note*. Unstandardized estimates. ^a–w^Corresponding coefficients are constrained to equality.

Estimates = means. Intercept = initial level at 2 months before relocation. Slope = change 2 months before–12 months after relocation.

### Stressors

#### Relocators

Starting with the results of the univariate growth curves for relocators, we found that a random latent basis model best fit the data for general stress with different participants experiencing different starting points and non-linear patterns of change over time. Next, we examined changes in stress in domains related to both partners’ careers. Relocators’ reports of stress related to starting a new job/school best fit a fixed latent basis model with a significant negative slope. This signifies that everyone followed the same non-linear pattern over time with stress decreasing from baseline (*M* = 4.52) to 3-months (*M* = 4.07), relatively remaining stable until 6-months (*M* = 4.11), decreasing at 9-months (*M* = 3.75), and increasing again 12-months (*M* = 4.04) post-relocation. However, a random intercept model best fit the data for stress related to their partner’s job/school (i.e., the accompanying partner’s job/school), signifying stability over time.

Further, we examined stress related to changes in participants’ social networks. Stress related to moving away from family changed in a non-linear pattern and best fit a fixed quadratic model with a significant negative slope and positive quadratic estimate. This stressor declined from baseline (*M* = 4.17) to 3-months (*M* = 3.79) but remained relatively stable from 3-months to 6-months (*M* = 3.84), 9-months (*M* = 3.88), and 12-months (*M* = 3.74) post-relocation. In contrast, we found a random intercept model best fit stress related to losing social ties, showcasing stability over time. A random slope model best fit the data for stress related to moving near family and establishing new social ties, although the slopes for these models were not significant, and these stressors did not change over time.

Next, examining changes in relocators’ stress related to their living arrangements, a fixed quadratic model best fit the data for stress related to finding a new place of residence and settling into a new home, with both stressors having significant negative slopes and the former having a significant positive quadratic estimate. Stress related to finding a new residence declined from baseline (*M* = 4.72) to 3-months (*M* = 4.28), and from 3-months to 6-months (*M* = 3.98), but remained relatively stable from 6-months to 9-months (*M* = 3.95) and 12-months (*M* = 3.95) post-relocation. Similarly, stress related to settling into a new home declined from baseline (*M* = 3.90) to 3-months (*M* = 3.68), remained stable from 3-months to 6-months (*M* = 3.61), declined from 6-months to 9-months (*M* = 3.47), and increased from 9-months to 12-months (*M* = 3.65) post-relocation, reaching stress levels similar to the first follow-up. We found that a fixed slope model with a significant negative slope best fit the data for changes in standards of living. This signifies that relocators began the study with varying levels of stress, but the stress related to changes in standards of living significantly decreased at the same rate for all relocators over time.

Finally, we examined relocators’ stress related to logistics. A random intercept model best fit the data for stress related to navigating a new culture/language, indicating that while some relocators started off with higher or lower levels of stress than others, there was no change in these stressors over time. A random slope model best fit the data for stress related to relocation finances and administrative tasks with significant negative slopes. This indicates that while participants started with varying levels of stress related to these variables, the rate at which their stress changed over time differed across individuals, highlighting individual differences in the adjustment process.

#### Accompanying Partners

Turning to accompanying partners’ reports of stressors, we found a fixed latent basis model for general stress with a significant negative slope, indicating a similar non-linear pattern for all participants with stress declining from baseline (*M* = 4.37) to 3-months (*M* = 3.51), 3-months to 6-months (*M* = 3.11), but relatively no change from 6-months to 9-months (*M* = 3.21) and 12-months (*M* = 3.16) post-move. Next, we examined career related stressors. A random latent basis model with a significant negative slope best fit data for stress related to accompanying partners starting a new job/school, indicating non-linear change. Accompanying partners’ reports of stress related to their partner’s career (i.e., relocators’ career) best fit a random slope model with a non-significant slope, highlighting no overall change in this trajectory.

Next, we examined stress related to changes in accompanying partners’ social networks. Stress related to moving away from family, losing social ties, and establishing new social ties exhibited no change over time and best fit a random intercept model. Although stress related to moving near family best fit a random latent basis model, the slope of this model was not significant, and this stressor remained stable. In contrast, stress related to partners’ living arrangements changed over time. Participants’ reports of stress related to finding a new place of residence best fit a random slope model and decreased over time at different rates for different participants. We found non-linear patterns of change for stress related to settling into a new home and changes in standards of living which best fit random latent basis models with significant negative slopes. Finally, we examined stress related to logistics over time. Although stress related to administrative tasks exhibited no change over time and best fit a random intercept model, stress related to navigating a new culture and finances best fit a fixed slope model with significant negative slopes indicating a decrease in stress for all participants at the same rate.

#### Dyadic Latent Growth Curves

**Figure 1.**
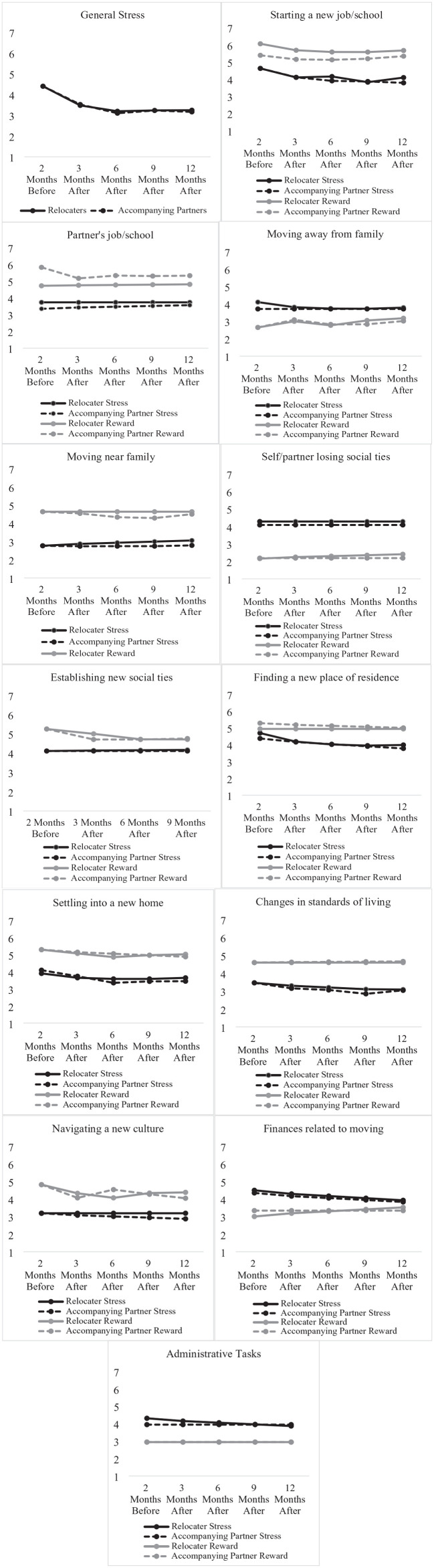
Trajectories of estimated means from dyadic growth curve models of relocators’ and accompanying partners’ stressors and rewards during the first year of relocation. *Note.* For some variables, the trajectories of both partners were not statistically different and were constrained to equality. For these variables, the trajectories of both partners are represented by one line.

To determine whether the trajectories of stressors are significantly different between relocators and accompanying partners, we added equality constraints to the intercepts and slopes of partners’ trajectories (see solid black and gray lines in Figure 1). We found that relocation role significantly moderated the results for half of the stressors, including partner’s job/school, moving away from family, losing social ties, finding a new place, settling into a new home, relocation finances, and administrative tasks. More specifically, relocators started off having higher stress levels related to their partner’s job/school, losing social ties, and relocation finances compared to accompanying partners. Although stress related to moving away from family changed non-linearly for relocators, declining from baseline to 3 months post-move and remaining stable until the last follow-up, this stressor remained stable for accompanying partners. Stress related to finding a new place changed non-linearly for relocators, declining from baseline through 6-months post-move then stabilizing. For accompanying partners, however, this stressor decreased over time at different rates for different participants. In contrast, settling into a new home started off being more stressful for accompanying partners compared to relocators, although both partners’ stress declined over time. Relocators experienced greater stress related to administrative tasks and their stress decreased, while accompanying partners’ stress was stable over time. The trajectories of the remaining stressors were not significantly different between partners, even if in some cases they had different best-fitting models (i.e., there was no difference between intercepts when set at the first and last time points).

### Rewards

#### Relocators

Next, we estimated univariate growth curves for rewards reported by relocators. Starting with rewards related to both partners’ careers, a fixed quadratic model best fit rewards associated with relocators starting a new job, with a significant negative slope and positive quadratic estimate indicating a decline from baseline (*M* = 6.10) to 3-months (*M* = 5.58), an increase from 3-months to 6-months (*M* = 5.72), and relatively no change from 6-months to 9-months (*M* = 5.64) and 12-months (*M* = 5.66) post-move. Although a random slope model best fit data for rewards related to their partner’s job/school, the slope was not significant indicating no overall change.

Next, we examined rewards related to changes in social networks. We found that rewards associated with moving near family did not change over time and best fit a random intercept model. In contrast, rewards associated with losing social ties best fit a fixed slope model with a significant positive slope, indicating a uniform increase in rewards for all relocators. A random latent basis model best fit rewards associated with moving away from family and establishing new social ties. This indicates that relocators showed varied patterns, but, on average, rewards from moving away from family increased, while those from forming new social ties decreased over time.

Rewards related to changes in living arrangements mostly remained stable with a random intercept model best-fitting data for finding a new place and changes in standards of living. However, a random latent basis model best fit rewards associated with settling into a new home. Relocators showed varied patterns, but on average rewards decreased over time.

Examining rewards related to adjustment, we found that rewards associated with navigating a new culture best fit a fixed latent basis model with a significant negative slope, declining from baseline (*M* = 4.99) to 3-months (*M* = 4.54), from 3-months to 6-months (*M* = 4.10), but increasing from 6-months to 9-months (*M* = 4.36) and from 9-months to 12-months (*M* = 4.49) post-relocation. Rewards associated with relocation finances best fit a fixed slope model with a significant positive slope, indicating a uniform increase in rewards for all relocators. However, rewards for administrative tasks remained stable and best fit a random intercept model.

#### Accompanying Partners

We then estimated univariate growth curves for accompanying partners. Rewards related to both partners’ careers changed non-linearly over time. More specifically, rewards related to accompanying partners starting a new job/school best fit a fixed quadratic model with a significant negative slope and a positive quadratic estimate indicating a decline from baseline (*M* = 5.46) to 3-months (*M* = 5.06), no change from 3-months to 6-months (*M* = 5.07), an increase from 6-months to 9-months (*M* = 5.30), and no change from 9-months to 12-months (*M* = 5.39) post-move. Rewards related to their partner’s career best fit a fixed latent basis model and exhibited similar trajectories across all accompanying partners and had a significant negative slope, declining from baseline (*M* = 5.85) to 3-months (*M* = 5.19), increasing from 3-months to 6-months (*M* = 5.30), remaining relatively stable from 6-months to 9-months (*M* = 5.26), and increasing from 9-months to 12-months (*M* = 5.36) post-move.

Examining rewards related to changes in social networks, rewards related to moving away from family best fit a fixed latent basis model with a significant positive slope and exhibited similar trajectories across all accompanying partners, increasing from baseline (*M* = 2.62) to 3-months (*M* = 3.04), decreasing from 3-months to 6-months (*M* = 2.86), increasing from 6-months to 9-months (*M* = 2.96), and increasing from 9-months to 12-months (*M* = 3.06) post-move. However, it is important to note that the means for this variable were low overall across the time points. Next, rewards related to losing social ties best fit a random slope model, but the slope was not significant indicating no average change, with individual participant slopes flowing in all directions. Rewards related to moving near family and establishing new social ties best fit a random latent basis model with different participants experiencing different patterns of change over time. Rewards related to establishing new social ties significantly decreased on average over time.

Rewards related to living arrangements including finding a new place, settling into a new home, and changes in standards of living all fit a random slope model. However, only the slope for settling into a new home was significant and declined over time. Finally, examining logistics, rewards related to relocation finances and administrative tasks best fit a random intercept model and remained stable. However, rewards from navigating a new culture best fit a random latent basis model with different participants experiencing different patterns of change over time and rewards decreased on average over time.

#### Dyadic Latent Growth Curves

We added equality constraints to the intercepts and slopes of partners’ trajectories of rewards to determine whether they were moderated by relocation role (see dashed black and gray lines in Figure 1). We found that relocation role moderated the results for rewards associated with starting a new job, partner’s job/school, losing social ties, finding a new place, settling into a new home, and relocation finances. More specifically, although rewards for staring a new job/school changed non-linearly for both partners, relocators started off experiencing more rewards 2 months before relocation. In contrast, accompanying partners started off experiencing more rewards about their partner’s (i.e., the relocator’s) job/school and experienced a decline in these rewards over time, while relocators experienced stability. Further, relocators experienced an increase in rewards related to losing social ties over time, while accompanying partners experienced stability. Accompanying partners also started off having more rewards related to finding a new place. Although both partners started off with the same level of rewards related to settling into a new home and these rewards declined at a similar rate over time, this change was non-linear for relocators and linear for accompanying partners. Finally, relocators started off experiencing fewer financial rewards 2-months before relocation, but these rewards increased over time while they remained stable for accompanying partners. The differences between partners’ trajectories for the remaining variables were not significant.

### Gender

A strength of our sample is that we had relatively equal numbers of men and women, balanced across relocating and accompanying partner roles. This allowed us to include relocator gender as a predictor of relocators’ own and their partners’ best-fitting unconstrained univariate growth curve for each variable (see Table 5 for model estimates). Given that 91.2% of our sample were in mixed-gender relationships, we only report the results for relocator gender predicting the trajectories. Being a relocating man (vs. relocating woman) predicted lower initial levels of stress related to establishing new social ties and relocation finances, and greater decrease in stress related to finances. Gender did not significantly predict the trajectories of any of the other stressors. We also found that women accompanying partners (vs. men accompanying partners) had higher initial levels of rewards associated with starting a new job/school. Relocating men (vs. relocating women) had higher initial rewards related to losing social ties, and both relocating men and accompanying women started out with more rewards related to finances and administrative tasks.

**Table 5.** Relocator Gender Predicting Trajectories of Stressors and Rewards During Partnered Relocation.

Variable	Stressors				Rewards			
	Relocators		Accompanying Partner		Relocator		Accompanying Partner	
	Intercept	Slope	Intercept	Slope	Intercept	Slope	Intercept	Slope
General stress	−.28	.03	.17	−.00	—	—	—	—
Careers								
Starting a new job/school	−.42	—	−.18	−.04	−.15	—	**.52**	−.04
Partner’s job/school	.01	—	−.04	—	−.27	.02	.03	—
Social Networks								
Moving away from family	−.13	.01	.40	—	.33	−.03	.36	—
Moving near family	.08	−.03	.27	.03	−.10	—	.44	—
Self/partner losing social ties	−.24	—	.05	—	**.43**	—	.28	—
Establishing new social ties	−**.56**	.02	.18	—	−.02	—	.07	—
Living Arrangements								
Finding a new place of residence	−.06	.03	.24	−.00	−.18	—	.36	−.02
Settling into a new home	.03	.02	.31	−.00	−.33	.02	.16	—
Changes in standards of living	.04	.01	.09	—	.39	—	.42	−.01
Adjustment								
Navigating a new culture	.19	—	.38	—	−.26	—	−.46	.03
Finances related to moving	−**.79**	**.05**	.06	−.03	**.64**	—	**.65**	—
Admin tasks	−.08	−.00	.08	—	**.82**	—	**.66**	—

*Note*. Unstandardized estimates. Gender (1 = *woman*, 2 = *man*) is only entered as a predictor of random (vs. fixed) intercepts and slopes. Intercept = initial level 2 months before relocation. Slope = change 2 months before – 12 months after relocation. Bolded coefficients are significant at *p* < .05.

### Moving After the Height of COVID-19

For couples who relocated after (*n* = 99; vs. before, *n* = 107) the height of the pandemic, accompanying partners started out with less stress related to navigating a new culture, while relocators showed a smaller increase in their perception of rewards for their partner’s job or school. Accompanying partners who moved after the rollout of the vaccines started out with fewer rewards for moving away from family, losing social ties, administrative tasks, and had a greater decrease in rewards related to navigating a new culture (see Table S3).

### Moving Internationally

Relocators who moved internationally (vs. domestically) started out with more stress regarding starting a new job and their partner’s job and administrative tasks, while accompanying partners who moved internationally had greater initial stress for establishing new social ties, navigating a new culture, and administrative tasks. Accompanying partners moving internationally also had a larger decrease in their general stress and stress related to finding a new place. Moving on to rewards among those moving internationally, relocators began with more rewards for moving near family, while both partners started with fewer rewards for losing social ties and administrative tasks. Accompanying partners showed a larger decrease in rewards for finding a new place and less increase in rewards related to changes in standard of living. They also started with more rewards for navigating a new culture but experienced a larger decrease in these rewards over time (Table S4).

### Socioeconomic Status

Relocators who had higher SES started out with less stress about their partner’s job and school and navigating a new culture. Accompanying partners with higher SES started out with less stress about losing social ties and finances, more rewards for establishing new social ties and administrative tasks and had a smaller decrease in rewards related to navigating a new culture (Table S5).

### Relationship Quality

Both relocators and accompanying partners had a larger decrease in their general stress if they reported being more satisfied with their relationship. Satisfied relocators and accompanying partners both started out with lower stress related to their careers, social networks, changes in standards of living, and navigating a new culture. Satisfied relocators had a smaller increase in stress for establishing new social ties and smaller decrease in stress related to administrative tasks. Satisfied accompanying partners started out with less stress for administrative tasks and showed a greater decrease in stress related to starting a new job. Further, when relocators were more satisfied, they started out with less rewards for losing social ties. Satisfied relocators and accompanying partners started out with more rewards regarding their careers, establishing social ties, and living arrangements. Satisfied accompanying partners started out with fewer rewards for moving away from family and losing social ties, more rewards for moving near family, and had a larger decrease in rewards related to finding a new place (Table S6).

## Discussion

Consistent with prior research documenting the stressors associated with moving (Brown, 2008; Harris, 2004; Harvey, 1998; McNulty, 2012), participants in our study found domains such as financial management, administrative tasks, and loss of social networks to be more stressful than rewarding at all time points. However, stress levels across all domains were generally moderate (i.e., not above the mid-point), even in the early stages of the move. Reinforcing the value of considering relocation rewards alongside stressors, we also found that certain domains—particularly those related to both partners’ careers, new social connections, and living arrangements—were rated as more rewarding than stressful. Several domains, such as those related to both partners’ careers, finding a new place of residence, and settling into a new home, were experienced as *highly* rewarding. These findings contribute a more balanced perspective to a literature that has historically focused on the burdens of relocation (Sterle et al., 2018). Overall, while relocation is often stressful, it can also be a time of growth, and focusing on its rewards may help couples find fulfillment in the transition.

### Trajectories of Stressors and Rewards

Many stressors declined over the first year following relocation, including general stress, along with stress related to both partners’ careers, moving away from family, living arrangements, and move-related logistics. These patterns suggest a process of adaptation whereby disruptive elements, such as determining new living arrangements or dealing with administrative hurdles, become more manageable as couples establish routines and gain familiarity with their surroundings. This pattern aligns with models of acute stress that highlight temporary disruption followed by recovery as people regain control over their environment (Lucas et al., 2003). In contrast, some rewards declined over time—especially those associated with novelty such as starting a new job and adjusting to a new culture. Meanwhile, rewards that were slower to emerge, such as financial stability or relief from previous social obligations, increased slightly as time progressed.

While several domains showed meaningful change, others remained stable. Stress related to a partner’s career, social network disruptions, and navigating a new culture or language remained stable over time. Similarly, rewards related to moving near family, finding housing, changes in standards of living, and administrative tasks remained stable. This stability may reflect individual differences between people and couples, such as their personality, the amount of work–life conflict they experience, or the specific coping strategies they use, which can make it challenging to find a single average trajectory. That many stressors and rewards remained stable, despite the magnitude of the disruption to partners’ lives, where familiar routines, patterns, and social ties needed to be reconfigured, suggests a degree of resilience and perceived control in navigating the transition (Keeton et al., 2008).

### Differences and Similarities in Trajectories Across Relocation Roles

Some notable differences emerged based on whether individuals initiated or supported the move. Relocators generally experienced more stress prior to the move across several domains including moving away from family, losing social ties, finding housing, finances, and administrative tasks, compared to accompanying partners. They also reported greater early rewards related to starting their new position. Conversely, accompanying partners experienced more rewards related to their partner’s job, losing social ties, finding housing, and finances prior to relocation. These role-based differences may be shaped by the relocator’s feelings of responsibility or guilt for causing the disruption to their partners’ lives and careers (Righetti et al., 2020), and the accompanying partner’s decision to sacrifice by prioritizing their partner’s aspirations over their own. Over time, however, many of these differences diminished as accompanying partners reestablished their careers and both partners adapted to the transition.

Despite these distinctions, partners experienced similar trajectories in many domains. This similarity in partners’ experiences may seem counterintuitive given the different circumstances relocators and accompanying partners face and past findings showing that relocators report being more stressed about their jobs (e.g., Harvey, 1998). However, partners typically experience similar environmental changes (e.g., navigating unfamiliar settings and changing social networks), share similar daily experiences, concerns, and goals (Fitzsimons et al., 2015), and draw on overlapping support systems. These similarities were likely overlooked in prior research on relocation that focused on individual-level analyses rather than taking a dyadic perspective.

### Gender Differences in Trajectories of Stressors and Rewards

We also examined whether men and women experienced different stressors and rewards during relocation. For the most part, gender differences were relatively limited. Unlike earlier studies emphasizing women’s heightened stress from taking on more responsibilities (Harris, 2004; Mäkelä et al., 2017), our sample—which included many more accompanying men and relocating women than previous studies (Harvey, 1998; McNulty, 2012)—showed few consistent gender effects. Nonetheless, some distinctions emerged. Relocating men reported lower pre-move stress about finances and establishing new social ties, and their financial stress declined more sharply over time. They also found aspects like losing social ties, finances, and administrative tasks more rewarding than relocating women. As non-traditional couples (i.e., those with women relocators) challenge workplace norms, relocating women may feel both empowered by career opportunities and anxious about financial burdens, especially given persistent gender pay disparities (International Labour Organization, 2022). Accompanying women reported more pre-move rewards related to starting a new position, finances, and administrative tasks than accompanying men. Women are often socialized to prioritize interpersonal relationships and community involvement (Lopata, 2006), which may make new connections more fulfilling. That these financial differences emerge even among couples with non-traditional gender dynamics (i.e., women relocators and men accompanying partners) highlights how deeply ingrained gendered expectations around money and responsibility may continue to shape relocation experiences.

### Contextual and Relational Covariates

Beyond examining average patterns across the sample, we explored how contextual and relational factors shaped couples’ relocation experiences, revealing several important distinctions. Accompanying partners who moved after the height of the COVID-19 pandemic reported fewer stressors and rewards. Notably, the pandemic did not amplify relocation-related stress, suggesting that our findings may generalize to non-pandemic contexts. For instance, accompanying partners experienced less stress around cultural adjustment, perhaps due to improved institutional support, clearer travel guidelines, or greater openness after vaccines became widely available. However, perceived rewards declined more sharply, possibly due to higher expectations or quicker loss of novelty in the post-pandemic era. For example, accompanying partners who moved after the vaccine rollout reported fewer initial rewards for losing social ties, suggesting that time apart from loved ones may have felt more costly after prolonged periods of social isolation during the pandemic. The pandemic may have heightened the value of existing relationships, making the trade-offs of relocation feel less worthwhile.

Couples who moved internationally experienced more initial stress than those who moved domestically, especially around job transitions, social integration, cultural adjustment, and administrative demands. These challenges were especially pronounced for accompanying partners, suggesting that international moves may impose greater psychological and logistical burdens on accompanying partners. Interestingly, while international relocators initially reported more rewards related to moving near family and navigating a new culture, these benefits faded more rapidly over time, possibly as the realities of long-term adaptation set in.

Higher SES appeared to buffer against some relocation-related stress and increase perceptions of some rewards. Relocators with higher SES reported less stress related to their partner’s career and cultural adjustment, while accompanying partners experienced fewer financial concerns and more rewards, especially in navigating bureaucracy and building new social ties. These advantages may reflect access to greater financial resources, social capital, or institutional support, which can ease the transition and enhance the perceived gains.

Finally, more satisfied couples tended to fare better across multiple domains. Both relocators and accompanying partners with higher relationship satisfaction reported lower initial stress, steeper declines in general stress, and more consistent rewards from the move, particularly in areas related to careers, social networks, and home life. These findings suggest that strong relational foundations may help couples reframe stressful experiences more positively and maintain a sense of shared purpose and optimism throughout the transition. Together, these findings highlight that relocation is not a uniform experience, but one that is shaped by timing, geography, socioeconomic resources, and relationship dynamics, all of which influence how couples adapt and find meaning in the move.

### Theoretical and Practical Implications

Our results showcase how relocating couples appraise their environment and adjust their perceptions of how stressful or rewarding different domains are over time. The decline in most stressors suggests that partners manage distress by adjusting to their new environment and engaging in effective coping strategies. In many domains, couples viewed the move as more rewarding than stressful. Importantly, stress and reward emerged as distinct but co-occurring dimensions. While general trends pointed to decreasing stress and increasing rewards, some domains—such as starting a new job or finding housing—were initially both highly stressful and rewarding, suggesting that some experiences may be characterized by ambivalence. These patterns align with frameworks that conceptualize positive and negative evaluations as independent (Righetti et al., 2020; Watson et al., 1988). Focusing solely on stress would have obscured this complexity. Theoretically, our results highlight the value of multidimensional approaches that capture the simultaneous gains, losses, excitement, and strain that often define relocation experiences.

Our findings also highlight the complexity of individual experiences, as not all stressors and rewards during relocation followed the same pattern. This underscores the need to consider individual differences and couple-level dynamics in adaptation, as emphasized by the vulnerability–stress–adaptation (VSA) model (Karney & Bradbury, 1995). The VSA model posits that couples’ responses to stressors such as relocation depend on enduring traits (e.g., personality) and adaptive processes used to manage stress (e.g., communication) that influence relationship quality and stability (Karney & Neff, 2013). Prior research on work–family transitions shows that vulnerabilities like depression can hinder adjustment and relationship quality (Trillingsgaard et al., 2014), reinforcing the importance of applying the VSA model to understand how couples navigate relocation.

Our findings have practical implications for couples navigating relocation. Relocation often involves multiple stressors, making thorough pre-move preparation essential. Discussing anticipated challenges—such as job changes, finances, social networks, and administrative tasks—can help couples set realistic expectations, develop effective coping strategies, and better support each other throughout the transition (Challiol & Mignonac, 2005). Seeking external support is also critical, especially for accompanying partners who may lack professional and social resources during the adjustment period (McNulty, 2012). This support can include reaching out to counseling services, joining social networks or support groups, and utilizing community resources tailored to relocation. Our work offers insights into which stressors are most acute and when rewards may be leveraged more effectively.

### Limitations and Future Directions

A notable strength of our study is that we followed both members of romantic couples—with a relatively balanced sample of men and women across relocating and accompanying partner roles—from before their move to a year post-relocation. However, we relied on single-item measures of stressors and rewards, limiting our ability to capture the specific sources of difficulty or satisfaction with each domain. Future research should adopt more granular measures or qualitative methods to better understand nuances. Additionally, our focus on domains that people find challenging during relocation identified in previous research (Munton, 1990) may have excluded other relevant areas, such as identity shifts, belonging, safety, or recreational opportunities, which could also shape couples’ relocation experiences.

Our sample may not have been representative of all relocating couples, as participants reported only moderate stress levels and were generally highly satisfied with their relationship, consistent with other dyadic samples (Park et al., 2021). This may reflect couples who are especially committed and responsive are better equipped to manage stress and recognize the rewarding aspects of the move. Our recruitment strategy also focused on individuals relocating for work or school, with most relocators moving for career or educational opportunities. As such, the experiences of those relocating due to financial instability, job loss, or family obligations—who face greater financial strain, job insecurity, or emotional burdens of caregiving—are likely underrepresented. These circumstances likely introduce qualitatively different stressors—such as financial hardship or caregiving demands—that fall outside the scope of our current study. Future research should aim to recruit more diverse samples, especially from lower socioeconomic backgrounds or those relocating out of necessity rather than choice, to better capture the full range of relocation experiences.

## Conclusion

In today’s globalized world, relocation is increasingly prevalent and presents many challenges and opportunities for growth. This study offers a nuanced and dynamic portrait of how romantic partners experience and adapt to these challenges and opportunities. By tracking both stressors and rewards across multiple life domains and over time, our findings demonstrate that while many relocation-related stressors diminish and some rewards grow, the process is far from uniform. The trajectories of stress and reward are shaped not only by partners’ relocation roles and gender, but also by relational, socioeconomic, and contextual factors. Our results underscore the importance of adopting a dyadic and multidimensional perspective on relocation—one that acknowledges not only the hardships but also the potential for growth and fulfillment in the midst of life upheaval. As more couples face geographic moves for work, our findings offer valuable insights into how they can anticipate, manage, and ultimately thrive through this complex life transition.

## Supplemental Material

sj-docx-1-psp-10.1177_01461672251355002 – Supplemental material for On the Move: Trajectories of Stressors and Rewards Among Relocating Couples Supplemental material, sj-docx-1-psp-10.1177_01461672251355002 for On the Move: Trajectories of Stressors and Rewards Among Relocating Couples by Hanieh Naeimi, Haeyoung Gideon Park, Matthew D. Johnson, Mariko L. Visserman, Rebecca Horne and Emily A. Impett in Personality and Social Psychology Bulletin

## Notes

1 We also asked participants about stress and reward related to changes to their children’s education and/or social ties. However, given that 76.6% of our sample did not have children, we were underpowered to run our analyses as planned and many of our models, including the dyadic growth curves, did not converge for this variable given the small sample. As such, we decided to omit discussing this variable in text. Information about model fit indices for this variable is available on the OSF, and mean values across time points are available in Table S2.
