# Supplementary material for: On the Move: Trajectories of Stressors and Rewards Among Relocating Couples
Source: Pers Soc Psychol Bull. 2025 Aug 3;52(9):2999–3016. doi: 10.1177/01461672251355002 (PMC13392154; doi:10.1177/01461672251355002)
Supplement: sj-docx-1-psp-10.1177_01461672251355002 – Supplemental material for On the Move: Trajectories of Stressors and Rewards Among Relocating Couples [file sj-docx-1-psp-10.1177_01461672251355002.docx]

**Online Supplementary Materials**

**Table S1**

*Results from paired sample t-tests comparing stressors and rewards at each time point* (*N* = 383)

| Variable | Baseline | 3 Months | 6 Months | 9 Months | 12 Months |
| --- | --- | --- | --- | --- | --- |
| **Careers** |  |  |  |  |  |
| Starting a new job/school | -8.40 (314)  *d* = 2.33 | -7.42 (268)  *d* = 2.51 | -8.45 (262)  *d* = 2.60 | -9.46 (239)  *d* = 2.55 | -8.04 (214)  *d* = 2.65 |
| Partner’s job/school | -12.07 (313)  *d* = 2.51 | -9.76 (293)  *d* = 2.54 | -8.45 (279)  *d* = 2.71 | -7.99 (261)  *d* = 2.59 | -8.51 (229)  *d* = 2.64 |
| **Social Networks** |  |  |  |  |  |
| Moving away from family | 8.27 (269)  *d* = 2.83 | 3.69 (260)  *d* = 2.82 | 5.71 (237)  *d* = 2.84 | 4.86 (210)  *d* = 2.84 | 3.17 (180)  *d* = 2.85 |
| Moving near family | -8.91 (185)  *d* = 2.95 | -8.72 (149)  *d* = 2.59 | -6.85 (155)  *d* = 2.87 | -5.84 (131)  *d* = 3.11 | -5.04 (117)  *d* = 3.18 |
| Self/partner losing social ties | 17.69 (351)  *d* = 2.35 | 13.86 (328)  *d* = 2.58 | 13.58 (310)  *d* = 2.54 | 13.43 (269)  *d* = 2.42 | 11.94 (244)  *d* = 2.59 |
| Establishing new social ties | -8.97 (373)  *d* = 2.34 | -4.07 (330)  *d* = 2.48 | -3.74 (314)  *d* = 2.75 | -4.26 (286)  *d* = 2.66 | -5.05 (251)  *d* = 2.68 |
| **Living Arrangements** |  |  |  |  |  |
| Finding a new place of residence | -4.68 (360)  *d* = 2.32 | -6.10 (329)  *d* = 2.52 | -6.10 (310)  *d* = 2.59 | -7.00 (284)  *d* = 2.51 | -6.76 (248)  *d* = 2.56 |
| Settling into a new home | -10.85 (368)  *d* = 2.23 | -11.76 (345)  *d* = 2.27 | -11.48 (323)  *d* = 2.31 | -10.73 (294)  *d* = 2.35 | -9.02 (258)  *d* = 2.54 |
| Changes in standards of living | -6.73 (344)  *d* = 2.86 | -8.45 (319)  *d* = 2.98 | -8.29 (292)  *d* = 2.96 | -8.67 (272)  *d* = 2.96 | -7.78 (233)  *d* = 3.06 |
| **Adjustment** |  |  |  |  |  |
| Navigating a new culture | -7.36 (222)  *d* = 2.58 | -4.96 (186)  *d* = 2.47 | -3.88 (181)  *d* = 2.48 | -3.71 (170)  *d* = 2.47 | -4.21 (156)  *d* = 2.63 |
| Finances related to moving | 8.96 (370)  *d* = 2.75 | 7.32 (339)  *d* = 2.93 | 5.23 (320)  *d* = 2.87 | 3.11 (297)  *d* = 2.77 | 2.15 (259)  *d* = 2.82 |
| Admin tasks | 10.37 (355)  *d* = 2.44 | 7.78 (337)  *d* = 2.77 | 6.78 (315)  *d* = 2.68 | 6.55 (289)  *d* = 2.56 | 5.84 (256)  *d* = 2.61 |

*Note*. Values outside parentheses are t statistics and values inside parentheses are degrees of freedom. All coefficients are significant *p* < .05. Standardized Cohen’s d estimates are reported. Negative estimates indicate that stressor values are lower than reward values.

**Table S2**

*Means and Standard Deviations of Stressors and Rewards from 2 Months Before to 12 Months After the Move* (*N* = 383)

|  | Stressors | | | | | Rewards | | | | | |  |
| --- | --- | --- | --- | --- | --- | --- | --- | --- | --- | --- | --- | --- |
| Variable | 2 Months Before | 3 Months After | 6 Months After | 9 Months After | 12 Months After | 2 Months Before | 3 Months After | | 6 Months After | 9 Months After | 12 Months After | |
| Changes to children’s education and social ties | 4.03 (2.11) | 3.75 (1.78) | 3.71 (1.72) | 3.93 (1.79) | 3.32  (1.90) | 4.38 (1.81) | 4.82 (1.65) | 4.45 (1.86) | | 4.77 (1.65) | 4.49 (1.81) |  |

*Note*. Values outside parentheses are means and values inside parentheses are standard deviations. Items are rated on a scale from 1 to 7.

**Table S3**

*Relocation Period Relative to COVID-19 Predicting Trajectories of Stressors and Rewards During Partnered Relocation*

|  | **Stressors** | | | | **Rewards** | | | |
| --- | --- | --- | --- | --- | --- | --- | --- | --- |
|  | **Relocators** | | **Accompanying Partner** | | **Relocator** | | **Accompanying Partner** | |
| Variable | Intercept | Slope | Intercept | Slope | Intercept | Slope | Intercept | Slope |
| General stress | -.02 | -.00 | -.10 | .01 | — | — | — | — |
| **Careers** |  |  |  |  |  |  |  |  |
| Starting a new job/school | .28 | — | -.06 | .00 | -.04 | — | -.13 | .01 |
| Partner’s job/school | .15 | — | -.06 | — | .30 | **-.06** | -.12 | — |
| **Social Networks** |  |  |  |  |  |  |  |  |
| Moving away from family | -.54 | .03 | -.32 | — | -.08 | -.01 | **-.76** | — |
| Moving near family | -.29 | -.00 | -.43 | .01 | -.14 | — | -.14 | — |
| Self/partner losing social ties | -.10 | — | -.28 | — | -.24 | — | **-.42** | — |
| Establishing new social ties | -.31 | .02 | -.20 | — | -.18 | — | -.24 | — |
| **Living Arrangements** |  |  |  |  |  |  |  |  |
| Finding a new place of residence | -.36 | .01 | -.18 | -.01 | -.27 | — | .04 | -.01 |
| Settling into a new home | -.24 | .00 | -.15 | -.01 | .19 | -.03 | -.02 | — |
| Changes in standards of living | -.47 | .02 | -.28 | — | -.09 | — | -.35 | .02 |
| **Adjustment** |  |  |  |  |  |  |  |  |
| Navigating a new culture | -.29 | — | **-.77** | — | -.23 | — | .24 | **-.07** |
| Finances related to moving | -.05 | .00 | .19 | -.03 | -.33 | — | -.30 | — |
| Admin tasks | -.22 | .01 | -.34 | — | -.22 | — | **-.45** | — |

*Note*. Unstandardized estimates. Relocation Period (1 = relocation occurred between the onset of COVID-19 [March, 15, 2020] and widespread vaccine availability [January 1, 2021]; 0 = relocation occurred either before COVID-19 onset or after vaccines became available) is only entered as a predictor of random (vs. fixed) intercepts and slopes. Intercept = initial level 2 months before relocation. Slope = change 2 months before – 12 months after relocation. Bolded coefficients are significant at *p* < .05.

**Table S4**

*International Relocation Predicting Trajectories of Stressors and Rewards During Partnered Relocation*

|  | **Stressors** | | | | **Rewards** | | | |
| --- | --- | --- | --- | --- | --- | --- | --- | --- |
|  | **Relocators** | | **Accompanying Partner** | | **Relocator** | | **Accompanying Partner** | |
| Variable | Intercept | Slope | Intercept | Slope | Intercept | Slope | Intercept | Slope |
| General stress | .40 | .02 | -.32 | **.09** | — | — | — | — |
| **Careers** |  |  |  |  |  |  |  |  |
| Starting a new job/school | **.82** | — | .65 | .04 | .24 | — | .01 | -.02 |
| Partner’s job/school | **.83** | — | .42 | — | .25 | -.02 | .09 | — |
| **Social Networks** |  |  |  |  |  |  |  |  |
| Moving away from family | -.01 | -.00 | .24 | — | -.25 | -.04 | -.33 | — |
| Moving near family | .47 | -.04 | -.21 | .01 | **.91** | — | -.02 | — |
| Self/partner losing social ties | .06 | — | .29 | — | **-.63** | — | **-.72** | — |
| Establishing new social ties | .32 | -.00 | **.50** | — | .16 | — | .24 | — |
| **Living Arrangements** |  |  |  |  |  |  |  |  |
| Finding a new place of residence | .23 | .01 | -.48 | **.08** | .08 | — | .11 | **-.07** |
| Settling into a new home | .14 | -.02 | -.45 | .03 | .04 | .01 | -.03 | — |
| Changes in standards of living | -.27 | .01 | .19 | — | .05 | — | .12 | **-.09** |
| **Adjustment** |  |  |  |  |  |  |  |  |
| Navigating a new culture | .54 | — | **.74** | — | .42 | — | **1.14** | **-.07** |
| Finances related to moving | .31 | -.01 | .47 | .00 | -.28 | — | -.33 | — |
| Admin tasks | **1.08** | -.03 | **.78** | — | **-.70** | — | **-.75** | — |

*Note*. Unstandardized estimates. International relocation (1 = international; 0 = domestic) is only entered as a predictor of random (vs. fixed) intercepts and slopes. Intercept = initial level 2 months before relocation. Slope = change 2 months before – 12 months after relocation. Bolded coefficients are significant at *p* < .05.

**Table S5**

*Perceived Socioeconomic Status Predicting Trajectories of Stressors and Rewards During Partnered Relocation*

|  | **Stressors** | | | | **Rewards** | | | |
| --- | --- | --- | --- | --- | --- | --- | --- | --- |
|  | **Relocators** | | **Accompanying Partner** | | **Relocator** | | **Accompanying Partner** | |
| Variable | Intercept | Slope | Intercept | Slope | Intercept | Slope | Intercept | Slope |
| General stress | -.10 | .00 | -.11 | .00 | — | — | — | — |
| **Careers** |  |  |  |  |  |  |  |  |
| Starting a new job/school | -.06 | — | .00 | -.00 | .02 | — | .07 | .00 |
| Partner’s job/school | **-.19** | — | -.01 | — | -.01 | .01 | .06 | — |
| **Social Networks** |  |  |  |  |  |  |  |  |
| Moving away from family | -.02 | -.00 | -.16 | — | .07 | -.00 | .11 | — |
| Moving near family | .05 | -.01 | .00 | .01 | -.10 | — | .11 | — |
| Self/partner losing social ties | -.11^+^ | — | **-.13** | — | .05 | — | .06 | — |
| Establishing new social ties | -.11 | .00 | -.12 | — | .01 | — | **.12** | — |
| **Living Arrangements** |  |  |  |  |  |  |  |  |
| Finding a new place of residence | -.10 | -.00 | -.02 | -.00 | .07 | — | .04 | -.00 |
| Settling into a new home | -.15 | .01 | -.04 | .00 | .08 | .00 | .08 | — |
| Changes in standards of living | .07 | -.01 | .02 | — | .05 | — | -.03 | .00 |
| **Adjustment** |  |  |  |  |  |  |  |  |
| Navigating a new culture | **-.17** | — | -.07 | — | .04 | — | -.04 | **.02** |
| Finances related to moving | -.12 | .00 | **-.21** | .01 | .06 | — | .11 | — |
| Admin tasks | -.08 | .01 | .04 | — | .08 | — | **.12** | — |

*Note*. Unstandardized estimates. Perceived Socioeconomic Status (range = 10) is only entered as a predictor of random (vs. fixed) intercepts and slopes. Intercept = initial level 2 months before relocation. Slope = change 2 months before – 12 months after relocation. Bolded coefficients are significant at *p* < .05.

**Table S6**

*Relationship Quality Predicting Trajectories of Stressors and Rewards During Partnered Relocation*

|  | **Stressors** | | | | **Rewards** | | | |
| --- | --- | --- | --- | --- | --- | --- | --- | --- |
|  | **Relocators** | | **Accompanying Partner** | | **Relocator** | | **Accompanying Partner** | |
| Variable | Intercept | Slope | Intercept | Slope | Intercept | Slope | Intercept | Slope |
| General stress | -.14 | **-.02** | -.18 | **-.03** | — | — | — | — |
| **Careers** |  |  |  |  |  |  |  |  |
| Starting a new job/school | **-.35** | — | **-.56** | **-.04** | **.42** | — | **.36** | .03 |
| Partner’s job/school | **-.55** | — | **-.37** | — | **.50** | -.02 | **.51** | — |
| **Social Networks** |  |  |  |  |  |  |  |  |
| Moving away from family | -.05 | -.02 | -.24 | — | -.36 | -.01 | **-.52** | — |
| Moving near family | **-.98** | -.01 | **-.59** | .01 | -.05 | — | **.40** | — |
| Self/partner losing social ties | **-.35** | — | -.11 | — | **-.29** | — | **-.37** | — |
| Establishing new social ties | .06 | **-.05** | **-.34** | — | **.36** | — | **.38** | — |
| **Living Arrangements** |  |  |  |  |  |  |  |  |
| Finding a new place of residence | -.13 | -.01 | -.02 | -.02 | **.29** | — | .01 | **.04** |
| Settling into a new home | -.29 | -.02 | -.20 | -.02 | **.38** | -.01 | **.27** | — |
| Changes in standards of living | **-.49** | .01 | **-.48** | — | **.33** | — | -.11 | **.04** |
| **Adjustment** |  |  |  |  |  |  |  |  |
| Navigating a new culture | **-.60** | — | **-.68** | — | .06 | — | .16 | -.01 |
| Finances related to moving | -.04 | -.02 | -.04 | -.00 | -.03 | — | -.15 | — |
| Admin tasks | -.07 | **-.03** | **-.36** | — | .02 | — | -.24 | — |

*Note*. Unstandardized estimates. Relationship quality (range = 7) is only entered as a predictor of random (vs. fixed) intercepts and slopes. Intercept = initial level 2 months before relocation. Slope = change 2 months before – 12 months after relocation. Bolded coefficients are significant at *p* < .05.
